# Curbing Malaria: A New Hope through Clustered Regularly Interspaced Short Palindromic Repeats (CRISPR) Technology

**Published:** 2017-12-30

**Authors:** Manjit Panigrahi, Ansuman Panigrahi

**Affiliations:** 1 Animal Genetics Division, Indian Veterinary Research Institute, Izatnagar, Bareilly, Uttar Pradesh, India; 2 Department of Community Medicine, Kalinga Institute of Medical Sciences, KIIT University, Bhubaneswar, India

## Dear Editor

The term genome refers to the total genetic composition of an organism or species. The normal genes transcribe and translate into functional gene products, which in turn bring about a normal phenotype. Genomics, the study of genomes has revealed the significant level of diversity in the human genome attributed not only to single nucleotide polymorphisms (SNPs) but structural variations as well. It also allows us to explore fundamental details at the molecular level. Using molecular techniques, researchers have easily identified many molecular markers within a given species’ genome. However, the application of such knowledge in medical science and treatment of diseases is still in its infancy (1). The main challenge in genomics today is to understand the role of molecular aberrations in various diseases and to apply such knowledge to control the diseases. The ability to perturb the genome in a precise and targeted fashion is crucial for understanding genetic contributions to biology and disease.

Recently, a new form of genome editing known as CRISPR (Clustered Regularly Inter-spaced Short Palindromic Repeats) has shown promising results. The CRISPRs are found in approximately 40% of sequenced eubacteria genomes and 90% of sequenced archaea (2). It allows researchers to make very precise changes to the genome in cells of various organisms. The CRISPR and Cas (CRISPR-associated) proteins function as the RNA-based adaptive immune system in bacteria and archaea (3) and CRISPR sequences give bacteria a physical record of the viruses that bacteria come across. The protein associated with CRISPR system i.e. Cas9 is programmed to find and bind to specific sites in the virus genome, directed by special guide molecules made of RNA (4, 5). Also, CRISPR can add or delete base pairs at specifically targeted DNA loci and have been used to cut as many as five genes at once (6). By delivering the Cas9 protein and appropriate guide RNAs into a cell, the organism’s genome can be cut at any desired location (7).

Malaria parasites are unicellular organisms residing inside the red blood cells. Although the genomes of many malaria parasites have been sequenced, the functions of approximately half of the genes are still unknown. Thus, editing genes in malaria parasite genomes is still inefficient (8). Recently through gene drive, a method for stimulating biased inheritance of particular genes to alter entire populations of organisms, CRISPR can be incorporated in mosquito populations to aid in malaria control (9, 10). Briefly, a package of genes including the CRISPR system i.e. RNA-guided Cas9 endonuclease and guide RNAs along with the malaria resistance gene (gene controlling the immune response of mosquitoes against malaria) will be inserted into the germline cells that produce sperm/egg. The RNA guided Cas9 endonuclease cuts the genome at desired site and the broken strands of DNA trigger the cell’s repair mechanisms. While repairing the damage to the original gene, both the resistance gene and the CRISPR system will be copied to it. Then these engineered mosquitoes carrying the resistance gene and the CRISPR system will be allowed to mate with other wild-type mosquitoes. Consequently, these desired genes will be preferentially inherited to the off-spring. The CRISPR genes would produce their molecules inside the offspring and again cut the original gene from the wild type to copy over the resistance gene and the CRISPR system. This way, the CRISPR system can effectively make the same edit in every generation and finally create a mosquito population inherently resistant to the plasmodium parasite.

The CRISPR technology is very young and thus need more exhaustive studies in order to thoroughly evaluate the utility of this system, including the potential for off-target effects and our ability to accurately predict the ecological consequences of these interventions. Nevertheless, researchers have high hopes for wiping out malaria from the world through this newer, highly efficient genetic approach.
